# PROTOCOL: Factors influencing the implementation of non‐pharmacological interventions for behaviours and psychological symptoms of dementia in residential aged care homes: A systematic review and qualitative evidence synthesis

**DOI:** 10.1002/cl2.1393

**Published:** 2024-03-21

**Authors:** Hunduma Dinsa Ayeno, Gizat M. Kassie, Mustafa Atee, Tuan Nguyen

**Affiliations:** ^1^ Quality Use of Medicines and Pharmacy Research Centre, Clinical and Health Sciences University of South Australia Adelaide South Australia Australia; ^2^ Department of Pharmacy Ambo University Ambo Ethiopia; ^3^ The Dementia Centre, HammondCare Osborne Park Western Australia Australia; ^4^ Sydney Pharmacy School, Faculty of Medicine and Health The University of Sydney Sydney New South Wales Australia; ^5^ School of Nursing and Midwifery, Centre for Research in Aged Care Edith Cowan University Joondalup Western Australia Australia; ^6^ Curtin Medical School, Faculty of Health Sciences Curtin University Bentley Western Australia Australia; ^7^ School of Health Sciences Swinburne University of Technology Melbourne Victoria Australia; ^8^ National Ageing Research Institute Melbourne Victoria Australia

## Abstract

This is a protocol for a Cochrane Review. The objectives are as follows. This paper aims to describe a protocol for a systematic review that will synthesise the qualitative evidence regarding factors influencing the implementation of non‐pharmacological interventions (NPIs) for behavioural and psychological symptoms of dementia (BPSD) management in residential aged care homes (RACHs). The planned systematic review aims to answer the research question: ‘What are the factors influencing the implementation of NPIs in the management of BPSD at RACHs?’. Additionally, the planned systematic review also aims to generate recommendations to guide stakeholders (e.g., clinicians and aged care staff) and policymakers in the implementation of NPIs for managing BPSD at RACHs.

## 1 BACKGROUND

### 1.1 The problem, condition, or issue

Behaviours and psychological symptoms of dementia (BPSD) are defined as ‘a group of symptoms of disturbed perceptive thought content, mood, or behaviour that include physical aggression, screaming, restlessness, agitation, wandering, culturally inappropriate behaviours, sexual disinhibition, hoarding, cursing and shadowing, anxiety, depression, hallucinations, and delusions’ (Kozman, 2006, p. 1). Within 5 years of dementia diagnosis, at least one type of BPSD is reported in 90% of individuals, with apathy, anxiety, and depression being the most prevalent symptoms (Steinberg, 2008). Although these symptoms may present individually, most of the time, two or more symptoms co‐occur in RACH residents living with dementia (Cerejeira, 2012).

BPSD are often a difficult and distressing element of dementia care as reported by caregivers (Tanya, 2016). Research evidence indicates that BPSD (e.g., agitation) was linked to a 44% increase in the cost of care in residential aged care homes (RACHs) (Burley, 2020). Moreover, worsened cognition and BPSD were persistently linked to an elevated risk of RACH placement for individuals with dementia (Toot, 2017). As a result, RACH residents with dementia experience lower well‐being scores, and higher unmet needs in comparison to those who reside in their own homes (Tew, 2021). For example, a study conducted in Norway revealed that individuals with dementia residing in RACHs had a reduced quality of life, higher psychotropic use, higher dependency on mobility aids, limited social interactions, limited exercise, and reduced exposure to sunlight in comparison to their home dwelling counterparts (Olsen, 2016).

### 1.2 The intervention

BPSD management strategies can be broadly categorised into two types: pharmacological and non‐pharmacological interventions (NPIs) (Alves, 2013). Pharmacological interventions refer to measures that use medication to prevent or treat the conditions (Maciel, 2019). In contrast, NPIs refer to a range of interventions that do not involve the use of medications (Castellano‐Tejedor, 2022), such as psychosocial interventions (e.g., cognitive behavioural therapy, reminiscence therapy) (Turner, 2005; White, 2017), physical activity interventions (e.g., exercise therapy), sensorial interventions (e.g., music therapy, light therapy, multisensory stimulation), and staff focused interventions (e.g., training sessions) (Cabrera, 2015). In this paper, the term NPIs refers to any non‐medicine or non‐drug therapy intervention strategy relevant to BPSD management.

In the absence of any risk to the resident living with dementia or caregivers, NPIs should be the first‐line treatment for BPSD rather than pharmacological interventions (AAGP, 2003; Frederiksen, 2020; Guideline Adaptation Committee, 2016; Mazza, 2022; NICE, 2018). Clinical trials have shown that NPIs are effective in reducing BPSD and enhancing the overall well‐being of RACH residents with dementia and their caregivers with a greater effect size than the modest effect obtained from the use of psychotropic medications (Berg‐Weger, 2017). Nevertheless, studies have shown that the implementation of NPIs is often inadequate in RACHs (Anderson, 2022; Ervin, 2014; van der Ploeg, 2012).

Factors that deter the implementation of NPIs for BPSD in RACHs refer to the various obstacles that can prevent the successful implementation of NPIs in this setting. These obstacles include, but are not limited to, organisational factors (e.g., lack of manpower and resources) (Cousins, 2017; Ervin, 2014; Janzen, 2013), staff characteristics (e.g., knowledge gap, lack of time, turnover), dementia resident characteristics (e.g., poor physical and cognitive functions, resident unavailability due to daytime sleep, what works for one doesn't work for another i.e., ‘one size does not fit all’) (Cohen‐Mansfield, 2012; Ervin, 2014) and lack of collaboration among care providers (O'Donnell, 2021). On the other hand, factors that facilitate NPIs implementation for BPSD refer to the various factors that can support the successful implementation of NPIs. These include staff‐related factors, such as collaboration among the care staff, adequate care time, and a sufficient number of staff (Hussin, 2021).

### 1.3 Why it is important to do this review?

Various systematic reviews have explored the efficacy of different NPIs such as, reminiscence therapy, social interaction interventions (Cho, 2023), exercise and distraction therapies (Robinson, 2006), music‐based interventions (de Oliveira, 2015; Goris, 2016), light exposure, staff training, night‐time activities, daytime activities (Wilfling, 2021), and sensory stimulation (Sella, 2022) for the management of BPSD. Additionally, a meta‐analysis of 20 studies showed that music therapy decreased anxiety and behavioural symptoms (Ueda, 2013). However, no systematic review focused on the factors affecting the implementation of NPIs, although the implementation of an intervention is as important as the efficacy of the intervention itself in achieving intervention success in the real world. This was despite the fact that several individual cross‐sectional studies have identified different factors hampering the implementation of NPIs including lack of familiarity with residents (Janzen, 2013), lack of staff training, experience, and confidence (Ervin, 2014; Hussin, 2021; Kolanowski, 2010; Sung, 2011), as well as insufficient staffing (Ervin, 2014; Hussin, 2021; Kolanowski, 2010; Lewis, 2005) and staff time constraints (Ervin, 2014; Garrido, 2021; Hussin, 2021; Kolanowski, 2010; Miller, 2021; Sung, 2011). Because the planned systematic review intends to identify both the root causes of inadequate implementation of NPIs in RACHs and the facilitators that enhance their implementation, it is imperative to target qualitative studies. This systematic review and qualitative evidence synthesis will be conducted as one of the studies under a Ph.D. project which aims to develop a co‐design intervention to optimise the management of BPSD in RACHs.

## 2 OBJECTIVES

This paper aims to describe a protocol for a systematic review that will synthesise the qualitative evidence regarding factors influencing the implementation of NPIs for BPSD management in RACHs. The planned systematic review aims to answer the research question: ‘What are the factors influencing the implementation of NPIs in the management of BPSD at RACHs?’. Additionally, the planned systematic review also aims to generate recommendations to guide stakeholders (e.g., clinicians and aged care staff) and policymakers in the implementation of NPIs for managing BPSD at RACHs.

## 3 METHODS

**Figure 1:**
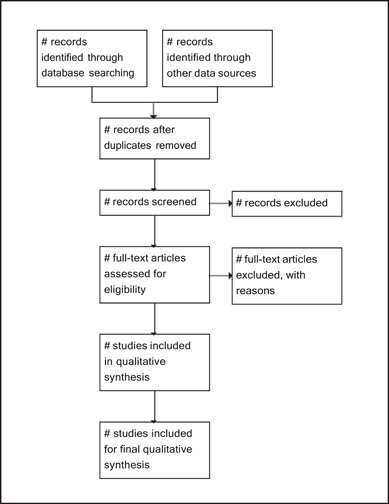
PRISMA flow chart for the study selection process.

The planned systematic review will include a comprehensive report on the search and selection of studies and the search results will be depicted in a flowchart that follows the Preferred Reporting Items for Systematic Reviews and Meta‐analyses (PRISMA) guidelines (Figure 1) (Page, 2021). The Joanna Briggs Institute's (JBI) approach to analysing qualitative evidence will be used (Aromataris, 2020). The theoretical domains framework (TDF) mapped to the capability, opportunity, and motivation behaviour change (COM‐B) model will be used to organise the findings into different categories (De Leo, 2021). Mapping COM‐B to TDF serves the purpose of bridging the gap between the broader conceptualisation of behaviour in COM‐B and the more specific domains outlined in TDF. This mapping allows for identifying the specific determinants and factors that influence a particular behaviour.

The TDF is an integrative framework developed from a synthesis of 33 psychological theories and serves as a vehicle to help apply theoretical approaches to interventions aimed at behaviour change (Phillips, 2015). It has 14 domains and has been developed to identify influences on health professional behaviour related to the implementation of evidence‐based recommendations (Atkins, 2017). These domains will be grouped into the COM‐B categories (De Leo, 2021). The TDF has been selected since it has a comprehensive coverage of the factors that contribute to the slow change of evidence into practice as well as its ability to shed light on barriers and facilitators of intervention implementation. Although these domains encompass factors related to health professionals' behaviour, according to Huijd et al. (2014), factors related to resident characteristics were categorised under the environmental context and resources. This systematic review and qualitative evidence synthesis will categorise the resident characteristics under the environmental context and resources. The systematic review protocol has been registered in PROSPERO (CRD42023388808, 11 February 2023).

### 3.1 Criteria for considering studies for this review

#### 3.1.1 Types of studies

Only qualitative data will be included for evidence synthesis to answer the research question of the planned systematic review. The sources of qualitative data include studies that employ various data collection methods including, but are not limited to, focus group discussions, interviews, qualitative surveys, ethnographies, and data analysis methods that encompass, but are not limited to, framework analysis, content analysis, and thematic analysis. Additionally, the qualitative components of the mixed methods studies will be included. However, qualitative studies with unsupported findings (i.e., findings not accompanied by illustrations or supporting evidence) will be excluded from the final synthesis. Studies will be selected if they report at least one implementation factor whether it is a barrier, facilitator, enabler, or related term irrespective of whether the word ‘implementation’ is present in the article or not. Implementation factors pertaining to the staff, families/relatives, volunteers, managers/leaders from their experience, that is, studies reporting the views and experiences of RACH staff, people with dementia, their families, and volunteers will be included. Only studies published in the English language and available in full text from the date of the inception of databases up until 31 December 2023 will be considered for inclusion. Articles published in a language other than English will be excluded because of resource constraints to retrieve these types of articles.

#### 3.1.2 Types of participants

The people living with dementia in RACHs, who are exhibiting BPSD will be included. Any study focusing on the general elderly population or a mixed population of people with dementia and the general elderly where there is no separate result presented for BPSD, and people with dementia who have no BPSD will be excluded.

#### 3.1.3 Types of interventions

The planned systematic review will include any NPI relevant to BPSD, neuropsychiatric symptoms, changed behaviours, responsive behaviours, or other synonyms for these terms in people with dementia. Examples of these interventions include music therapy, reminiscence therapy, validation therapy, meaningful activities, light therapy, multisensory stimulation, exercise therapy, a training programme for the care staff, person‐centred care, and Namaste care, to name a few. Other non‐pharmacological procedures that are not specifically used for BPSD such as surgery, radiation therapy, and imaging will not be included.

#### 3.1.4 Types of outcome measures

The outcomes of this systematic review will include a summary of findings regarding factors influencing the implementation of NPIs, drawing from the views and experiences of various stakeholders. These stakeholders include the RACHs staff (caregivers or personal care workers, nurses, nurse aides or assistants, allied health professionals, e.g., physiotherapists), physicians, volunteers, dementia residents' families, and the dementia residents themselves. The primary outcome will be a summary of the factors influencing the implementation of NPIs for BPSD at RACHs.

#### 3.1.5 Types of settings

In this review protocol, the setting RACHs which refers to residential facilities or long‐term care or nursing homes or housing for the elderly or residential homes or assisted living facilities or homes for the aged or aged care homes or halfway house(s) or group home(s) or retirement communities or sanatoriums or housing for older persons will be included. Any study focusing on other settings like hospitals, adult day care centres, homes, or community settings will be excluded. Overall, the type of studies, population, settings, and interventions discussed above are used as inclusion criteria.

### 3.2 Search methods for identification of studies

#### 3.2.1 Electronic searches

The search terms were developed with the assistance of the academic librarian at the University of South Australia. A preliminary search was conducted by entering the title of the systematic review into Google Scholar, and the Google search engine. The development of search terms for the research question focuses on four key aspects: the population (residents living with dementia), the phenomenon of interest (types of NPIs specifically used for managing BPSD), the context (RACHs), and the study type (qualitative studies).

Both Medical subject heading (MeSH) and keywords were used for each of these aspects.

An initial search was conducted on MEDLINE to locate articles about the research question. The keywords and index terms that appear in the titles, abstracts, and indexes of relevant articles were used to create a thorough search plan for MEDLINE. A set of articles that meet the inclusion criteria and are unambiguously eligible were chosen to authenticate the MEDLINE search plan. The search plan was translated, along with all the relevant MeSH terms, from MEDLINE to other databases such as EMCARE, Embase, CINAHL Complete, and APA Psych Info. The search in the selected databases has already been conducted up until 14 March 2023. Subsequent search updates will be performed up until 31 December 2023. A detailed listing of each database searched, and a line‐by‐line search is included in Supporting Information: Appendix 1. The OVID platform was used to search for articles in MEDLINE, EMCARE, Embase and APA Psych Info, while EBSCOhost was used to search for articles in CINAHL.

#### 3.2.2 Searching other resources

The relevant sources for grey literature including, Trove, and ProQuest dissertations, theses, and conference abstracts such as Alzheimer's Association International Conference as well as reference lists of included studies and any relevant systematic reviews will be identified. Due to time and resources constraints, contacting individuals and organisations for information about unpublished or ongoing studies will not be performed. Since any relevant published studies may be captured by the databases selected such as MEDLINE, Embase, PsycINFO, EMCARE, CINAHL, there is no plan for hand searching of a specific journal.

### 3.3 Data collection and analysis

#### 3.3.1 Description of examples of methods used in primary research

♦ Interviews are a popular qualitative method of data collection that involves asking questions to a single study participant or a group of participants in more natural settings. It can be conducted face‐to‐face, on the telephone, or through a video chat (Hancock, 2009).
♦ Focus groups are one type of qualitative method of data collection that involves inviting small groups of study participants to talk about a specific topic of interest. It employs a facilitator or moderator who will ask questions and guide the discussions where the participants are free to share their thoughts and experiences in their own words (Nyumba, 2018).
♦ Qualitative surveys are another type of qualitative data collection that uses open‐ended questions to gather participants' opinions, experiences, and perspectives on a certain topic of interest (Hancock, 2009).

#### 3.3.2 Selection of studies

The articles identified from the searches will be uploaded by the primary author (HA) into EndNote (version 20.2.1) to remove duplicates. After removing duplicates, the remaining articles will be imported into the JBI Database of Systematic Reviews and Implementation Reports (JBI SUMARI) software (Aromataris, 2020). The JBI SUMARI is primarily designed for qualitative systematic review and employs a meta‐aggregative method to synthesise qualitative evidence. One key characteristic of this approach is the ability to generate generalisable recommendations presented as a set of statements, which guide practitioners and policymakers (Aromataris, 2020). The initial stage will involve two reviewers (HA and GK) screening the title and abstract of the articles to exclude articles that are irrelevant using the JBI SUMARI tool. In the second stage, the full text of the remaining articles will be reviewed to include articles for final analysis. All disagreements in determining eligibility will be discussed between the two reviewers to reach an agreement. In case of ongoing disagreement, we will then discuss the disputed article(s) with a third author (TN or MA) to reach a consensus.

#### 3.3.3 Data extraction and management

A standardised tool will be used to extract information from the included qualitative studies (see Supporting Information: Appendix 2) within JBI SUMARI (Aromataris, 2020). Data extraction involves extracting details on the setting, participants, intervention, study methodology, and conclusions of the authors for each qualitative study. Data extraction will also include the list of factors influencing the NPI implementation along with their corresponding illustration. The primary author HA will conduct the data extraction process.

#### 3.3.4 Assessment of risk of bias in included studies

The methodological quality of the included articles will be assessed independently by two reviewers (HA and GK), using the standardised JBI SUMARI critical appraisal tool (Aromataris, 2020). The critical appraisal result will be presented in both narrative and tabular formats. The extraction of the list of factors influencing the implementation of NPIs, and their aggregation will be performed on all included articles, regardless of the outcomes of their quality assessment. The cut‐off point will be decided by the reviewers (GK and HA) before conducting a risk of bias assessment. All disagreements in determining the quality assessment scoring will be discussed between the two reviewers, to reach an agreement. In case of ongoing disagreement, we will then discuss the disputed article(s) with a third author (TN or MA) to reach a consensus.

#### 3.3.5 Data synthesis

A meta‐aggregation, a pragmatic synthesis method for qualitative studies that was developed at JBI, will be used to synthesise and summarise the practicalities and usefulness of the findings (Korhonen, 2013) in which each verbatim extracted finding will be accompanied by a participant's voice‐quoted directly with an assigned level of credibility. The degree of credibility will be determined based on the coherence between the finding and the corresponding illustration, classifying it as unequivocal, equivocal, and unsupported (Lockwood, 2015). The findings from the planned qualitative systematic review will be meta‐aggregated and presented with a set of statements in a table (Aromataris, 2020).

The findings will be organised into categories based on the similarity of their meanings. To establish evidence‐based practices, the categories will be merged to form a set of consolidated synthesised findings. The synthesis will include only unequivocal and credible findings (Aromataris, 2020). The TDF domains will be listed as categories in the JBI and each extracted finding (i.e., finding related to barrier or enabler or related term) will then be matched with these domains. Next, the meta‐aggregation diagram will be exported, and the resultant domains will be mapped to the COM‐B model.

#### 3.3.6 Summary of findings and assessment of the certainty of the evidence

To build assurance in the results of synthesis, the final synthesised findings will be assessed using the confidence of synthesised qualitative findings (ConQual) score and presented as a summary of the findings (Munn, 2014). The ConQual score is defined as the rating of confidence in the synthesised qualitative findings (Munn, 2014). The scoring process involves an initial ranking of the studies from high to very low based on their type. In this instance, qualitative studies are given a high rank, whereas expert opinions are ranked very low. Then, the scoring is given to the five yes/no questions (three congruity questions, one question related to researcher culture, and one question related to researcher influence). These questions measure how dependable the finding is. If the scoring has four to five yes responses, the study rank does not change (Aromataris, 2020). Otherwise, the study rank is downgraded by one level for two to three yes responses and by two levels for zero to one yes responses (Aromataris, 2020).

This initial ranking is followed by credibility scoring, in which the synthesised findings are categorised as unequivocal (finding followed by an illustration that has an undoubted association with it and cannot be challenged), credible (finding followed by illustration, but its association with the finding is unclear and open to criticism), and unsupported (finding is not supported by the accompanying illustration) (Munn, 2014). If the finding is unequivocal, the initial ranking of the study remains unchanged. However, the study is downgraded by one level for a mix of unequivocal/credible findings. For credible findings, the study is downgraded by two levels, and for a mix of credible/unsupported findings, the study is downgraded by three levels. Finally, for unsupported findings, the study is downgraded by four levels. After the findings are examined by applying the dependability and credibility rules, the total score is called the ConQual score (Munn, 2014).

The essential components of the summary will comprise the title, population, context, and phenomena of interest. Each synthesised finding will be presented in conjunction with the research type that informs it, as well as the dependability, credibility, and ConQual score (see Supporting Information: Appendix 3).

The summary of findings of the intended systematic review will include a description of the process of article inclusion using a PRISMA flowchart. The flowchart will provide a summary of the number of articles identified, screened, selected for retrieval, and included/excluded along with their reasons for exclusion. It will also detail the numbers appraised and included/excluded, as well as the numbers included in the qualitative synthesis. In addition, the results of the methodological quality of each included article will be presented using the JBI SUMARI critical appraisal checklist.

Furthermore, the description of the included articles based on the objective of the systematic review, which includes setting, country, methodology, study participant, and the phenomenon of interest will also be presented in a table. The last part of the summary of findings of the planned systematic review will include a meta‐aggregative overview flowchart. This flowchart will present four major components. The first component will consist of a list of a specified number of unequivocal and/or credible findings extracted from a specified number of the included studies, excluding unsupported findings as they will not be included in the meta‐synthesis. The second component will include a list of categories, each of which comprises one or more unequivocal and/or credible finding(s) extracted from the included studies. The third component will include a list of synthesised findings consisting of one or more categories along with a summary statement. Finally, the fourth component will include a list of recommendations that will inform the practitioners and policymakers. The findings of the review will be used as a basis for generating recommendations by synthesising and interpreting the collective insights derived from the evidence synthesis. In summary, the findings will be meta‐aggregated and presented in a table and diagram form.

## CONTRIBUTIONS OF AUTHORS

The review team includes Hunduma Dinsa Ayeno (M.Sc.), Dr. Gizat M. Kassie (Ph.D.), Dr. Mustafa Atee (Ph.D.), Assoc. Prof. Tuan Anh Nguyen (Ph.D., Associate professor). All the members of the review team possess expertise on the subject matter, systematic review methods, statistical analysis and information retrieval.

**Content**: Associate Professor Tuan Nguyen, Dr. Mustafa Atee, Dr. Gizat Kassie, and Mr. Hunduma Ayeno

**Systematic review methods**: Associate Professor Tuan Nguyen, Dr. Mustafa Atee, Dr. Gizat Kassie, and Mr. Hunduma Ayeno

**Data extraction**: Dr. Gizat Kassie, Mr. Hunduma Ayeno, Associate Professor Tuan Nguyen, and Dr. Mustafa Atee **Data synthesis**: Dr. Gizat Kassie, Mr. Hunduma Ayeno, Associate Professor Tuan Nguyen, and Dr. Mustafa Atee **Information retrieval**: Dr. Gizat Kassie, Mr. Hunduma Ayeno, Associate Professor Tuan Nguyen, and Dr. Mustafa Atee

All authors participated in conceptualising and designing the paper. HA drafted the paper with the contribution of GK, MA, and TN. All the authors were involved in reviewing the work and have given their approval for the final version to be submitted.

## DECLARATIONS OF INTEREST

M. Atee is a Research and Practice Lead at The Dementia Centre; a research, education, and consultancy arm of HammondCare, an independent Christian charity that auspices Dementia Support Australia (DSA) programmes – the Dementia Behaviour Management Advisory Service (DBMAS) and the Severe Behaviour Response Teams (SBRT). These programmes embrace and use multimodal, person‐centred psychosocial, and NPIs as part of their support strategies. The remaining authors have no conflict of interest.

### Preliminary timeframe

The finalised submission of the systematic review manuscript will be within 18 months of the protocol approval.

### Plans for updating this review

Full‐text screening is in progress. The review will be updated at least every month by HA.

## SOURCES OF SUPPORT

**Internal sources**

♦ Scholarship support, Australia
  No public, commercial or not‐for‐profit sectors provided any specific grant for this paper. However, Hunduma Ayeno is supported by the University of South Australia and Australian Government research training programme scholarship.
**External sources**
♦ New Source of support, Other
  No public, commercial or not‐for‐profit sectors provided any specific grant for this paper.

## Supporting information

Supporting information.
