# Supplementary material for: PROTOCOL: Factors influencing the implementation of non‐pharmacological interventions for behaviours and psychological symptoms of dementia in residential aged care homes: A systematic review and qualitative evidence synthesis
Source: Campbell Syst Rev. 2024 Mar 21;20(2):e1393. doi: 10.1002/cl2.1393 (PMC10958098; doi:10.1002/cl2.1393)
Supplement: Supplementary file 1 — Supporting information. [file CL2-20-e1393-s001.docx]

# Appendices

## Appendix 1: Search strategies

**Ovid MEDLINE(R) ALL <1946 to March 10, 2023>**

| **S. N** | **Search terms combined with Boolean logic using truncation** | **Results f rom 14**  **Mar 2023**  **search** |
| --- | --- | --- |
| 1 | exp residential facilities/ or housing for the elderly/ or exp long term care/ | 80,925 |
| 2 | (aged care or Assisted living or care facilit* or care home* or continu* care retirement communit* or convalescenc* home* or convalescenc* hospital* or geriatric* home* or group home* or half way house* or Halfway House* or health service* for the aged or home* for the aged or home* for the elder* or Housing for Older Person* or housing for the elder* or life care cent* or long term care or LTCF or nursing care cent* or nursing home* or old age* home* or old people* home* or Residential Care Institution* or residential facilit* or residential home* or residential institution or respite care or retirement cent* or Retirement Communit* or retirement home* or Sanatorium* or senior residence facilit* or Skilled nursing facilit* or Community living cent* or community cent* or adult family home* or memory care setting).ti,ab,kf. | 100,363 |
| 3 | dementia/ or alzheimer disease/ | 167,398 |
| 4 | (Alzheimer* or amentia* or dement* or pseudodementia).ti,ab,kf. | 272,978 |
| 5 | exp Sensory Art Therapies/ or spiritual therapies/ or Massage/ or exp Psychotherapy/ or sensory deprivation/ or Exercise Therapy/ or Occupational therapy/ or Recreation Therapy/ or Speech Therapy/ or Voice Training/ or Mind-body therapies/ | 340,796 |
| 6 | ("Acceptance and Commitment Therap*" or acoustic stimul* or activit* engagement or Acupressure or Acupuncture or anger management or animal assisted therap* or animal facilitated therap* or applied behavio* analys* or aroma therap* or Aromatherap* or art gallery or art treatment* or Art* therap* or auditory stimul* or Autogenic Training* or behavio* therap* or behavio* treatment* or Bibliotherap* or Cogniti* Remediation or cogniti* rehabilitation or Cogniti* Stimulation* or cogniti* therap* or cognitive enhancement* or Cognitive Intervention* or dance movement or Dance Therap* or Distraction or drama therap* or Environmental modiﬁcation* or Environmental Therap* or ergotherap* or Exercise Intervention* or exercise therap* or Exposure Therap* or eye movement desensitization or family intervention* or Family Therap* or garden* therap* or Gardening or group therap* or group treatment* or Guided Imagery or Hearing aid* or hearing rehabilitation* or horticultur* therap* or hypnos* or life story book* or light therap* or magic table* or Massage or Meditation* or Memory training* or Milieu Therap* or mindfulness or Montessori activit* or multicomponent intervention* or multisensory environment* or multisensory therap* or Music or nonpharmacolog* or non-pharmacolog* or occupation* therap* or Outdoor activit* or person cent* or Pet Therap* or physical activit* or Physical exercise* or physiotherapy or Prayer or Psychotherap* or reality therap* or recreation* therap* or relaxation therap* or Reminiscence* or sensory environment* or sensory stimul* or Simulated presence or snoezelen or social interaction* or Spaced retrieval or Speech Therap* or spiritual or therapeutic touch* or Tovertafel or validation therap* or Video Respite or Virtual Reality or visual feedback* or Yoga or namaste care).ti,ab,kf. | 561,700 |
| 7 | Qualitative research/ or interview/ or ethnology/ or focus groups/ or personal narratives/ or Interviews as Topic/ or grounded theory/ | 191,633 |
| 8 | (audiointerview* or content analys* or discourse analys* or ethnogeograph* or ethnograph* or ethnolog* or Focus Group* or grounded approach* or Grounded Theor* or Hermeneutic* or Interview* or Narrative Analys* or Naturalistic Inquir* or oral history as topic or Patient Reported or personal narrative* or Phenomenolog* or provider reported or Qualitative or Self Report or Structured Categor* or Thematic Analys* or theoretical sampling* or Unstructured Categor* or Videointerview).ti,ab,kf. | 813,674 |
| 9 | 1 or 2 | 137,417 |
| 10 | 3 or 4 | 290,856 |
| 11 | 5 or 6 | 767,000 |
| 12 | 7 or 8 | 853,716 |
| 13 | 9 and 10 and 11 and 12 | 620 |
| 14 | limit 13 to English language | 605 |

**Ovid Emcare <1995 to 2023 Week 09>**

| **S. N** | **Search terms combined with Boolean logic using truncation** | **Results f rom 14** |
| --- | --- | --- |

|  |  | **Mar 2023**  **search** |
| --- | --- | --- |
| 1 | Residential home/ or Nursing home/ or halfway house/ or assisted living facility/ or home for the aged/ | 27,535 |
| 2 | (aged care or Assisted living or care facilit* or care home* or continu* care retirement communit* or convalescenc* home* or convalescenc* hospital* or geriatric* home* or group home* or half way house* or Halfway House* or health service* for the aged or home* for the aged or home* for the elder* or Housing for Older Person* or housing for the elder* or life care cent* or long term care or LTCF or nursing care cent* or nursing home* or old age* home* or old people* home* or Residential Care Institution* or residential facilit* or residential home* or residential institution or respite care or retirement cent* or Retirement Communit* or retirement home* or Sanatorium* or senior residence facilit* or Skilled nursing facilit* or Community living  cent* or community cent* or adult family home* or memory care setting).ti,ab,kf. | 64,775 |
| 3 | dementia/ or Alzheimer disease/ | 72,769 |
| 4 | (Alzheimer* or amentia* or dement* or pseudodementia).ti,ab,kf. | 111,340 |
| 5 | exp Psychotherapy/ or Massage/ or sensory deprivation/ or sensory stimulation/ or auditory rehabilitation/ or kinesiotherapy/ or Occupational therapy/ or Recreation Therapy/ or Speech Therapy/ or Voice Training/ or phototherapy/ or animal assisted therapy/ or Virtual Reality Exposure Therapy/ or cognitive remediation therapy/ or meditation/ or aromatherapy/ or spiritual  healing/ | 131,241 |
| 6 | ("Acceptance and Commitment Therap*" or acoustic stimul* or activit* engagement or Acupressure or Acupuncture or anger management or animal assisted therap* or animal facilitated therap* or applied behavio* analys* or aroma therap* or Aromatherap* or art gallery or art treatment* or Art* therap* or auditory stimul* or Autogenic Training* or behavio* therap* or behavio* treatment* or Bibliotherap* or Cogniti* Remediation or cogniti* rehabilitation or Cogniti* Stimulation* or cogniti* therap* or cognitive enhancement* or Cognitive Intervention* or dance movement or Dance Therap* or Distraction or drama therap* or Environmental modiﬁcation* or Environmental Therap* or ergotherap* or Exercise Intervention* or exercise therap* or Exposure Therap* or eye movement desensitization or family intervention* or Family Therap* or garden* therap* or Gardening or group therap* or group treatment* or Guided Imagery or Hearing aid* or hearing rehabilitation* or horticultur* therap* or hypnos* or life story book* or light therap* or magic table* or Massage or Meditation* or Memory training* or Milieu Therap* or mindfulness or Montessori activit* or multicomponent intervention* or multisensory environment* or multisensory therap* or Music or nonpharmacolog* or non-pharmacolog* or occupation* therap* or Outdoor activit* or person cent* or Pet Therap* or physical activit* or Physical exercise* or physiotherapy or Prayer or Psychotherap* or reality therap* or recreation* therap* or relaxation therap* or Reminiscence* or sensory environment* or sensory stimul* or Simulated presence or snoezelen or social interaction* or Spaced retrieval or Speech Therap* or spiritual or therapeutic touch* or Tovertafel or  validation therap* or Video Respite or Virtual Reality or visual feedback* or Yoga or namaste care).ti,ab,kf. | 332,785 |
| 7 | Qualitative research/ or exp interview/ or ethnology/ or grounded theory/ or phenomenology/ or exp discourse analysis/ or exp  thematic analysis/ or exp content analysis/ or ethnography/ | 281,462 |
| 8 | (audiointerview* or content analys* or discourse analys* or ethnogeograph* or ethnograph* or ethnolog* or Focus Group* or grounded approach* or Grounded Theor* or Hermeneutic* or Interview* or Narrative Analys* or Naturalistic Inquir* or oral history as topic or Patient Reported or personal narrative* or Phenomenolog* or provider reported or Qualitative or Self Report or Structured Categor* or Thematic Analys* or theoretical sampling* or Unstructured Categor* or  Videointerview).ti,ab,kf. | 511,754 |
| 9 | 1 or 2 | 71,317 |
| 10 | 3 or 4 | 123,200 |
| 11 | 5 or 6 | 386,267 |
| 12 | 7 or 8 | 540,375 |
| 13 | 9 and 10 and 11 and 12 | 551 |
| 14 | limit 13 to english language | 539 |

**Embase Classic+Embase <1947 to 2023 March 10>**

| **S. N** | **Search terms combined with Boolean logic using truncation** | **Results f rom 14**  **Mar 2023**  **search** |
| --- | --- | --- |
| 1 | Residential home/ or Nursing home/ or halfway house/ or assisted living facility/ or home for the aged/ | 81,166 |
| 2 | (aged care or Assisted living or care facilit* or care home* or continu* care retirement communit* or convalescenc* home* or convalescenc* hospital* or geriatric* home* or group home* or half way house* or Halfway House* or health service* for the aged or home* for the aged or home* for the elder* or Housing for Older Person* or housing for the elder* or life care cent* or long term care or LTCF or nursing care cent* or nursing home* or old age* home* or old people* home* or Residential Care Institution* or residential facilit* or residential home* or residential institution or respite care or retirement cent* or Retirement Communit* or retirement home* or Sanatorium* or senior residence facilit* or Skilled nursing facilit* or Community living cent* or community cent* or adult family home* or memory care setting).ti,ab,kf. | 136,139 |
| 3 | dementia/ or Alzheimer disease/ | 354,791 |
| 4 | (Alzheimer* or amentia* or dement* or pseudodementia).ti,ab,kf. | 392,734 |
| 5 | exp Psychotherapy/ or Massage/ or sensory deprivation/ or sensory stimulation/ or auditory rehabilitation/ or kinesiotherapy/ or Occupational therapy/ or Recreation Therapy/ or Speech Therapy/ or Voice Training/ or phototherapy/ or animal assisted therapy/ or Virtual Reality Exposure Therapy/ or cognitive remediation therapy/ or meditation/ or aromatherapy/ or spiritual healing/ | 465,445 |
| 6 | ("Acceptance and Commitment Therap*" or acoustic stimul* or activit* engagement or Acupressure or Acupuncture or anger management or animal assisted therap* or animal facilitated therap* or applied behavio* analys* or aroma therap* or Aromatherap* or art gallery or art treatment* or Art* therap* or auditory stimul* or Autogenic Training* or behavio* therap* or behavio* treatment* or Bibliotherap* or Cogniti* Remediation or cogniti* rehabilitation or Cogniti* Stimulation* or cogniti* therap* or cognitive enhancement* or Cognitive Intervention* or dance movement or Dance Therap* or Distraction or drama therap* or Environmental modiﬁcation* or Environmental Therap* or ergotherap* or Exercise Intervention* or exercise therap* or Exposure Therap* or eye movement desensitization or family intervention* or Family Therap* or garden* therap* or Gardening or group therap* or group treatment* or Guided Imagery or Hearing aid* or hearing rehabilitation* or horticultur* therap* or hypnos* or life story book* or light therap* or magic table* or Massage or Meditation* or Memory training* or Milieu Therap* or mindfulness or Montessori activit* or multicomponent intervention* or multisensory environment* or multisensory therap* or Music or nonpharmacolog* or non-pharmacolog* or occupation* therap* or | 783,498 |

|  | Outdoor activit* or person cent* or Pet Therap* or physical activit* or Physical exercise* or physiotherapy or Prayer or Psychotherap* or reality therap* or recreation* therap* or relaxation therap* or Reminiscence* or sensory environment* or sensory stimul* or Simulated presence or snoezelen or social interaction* or Spaced retrieval or Speech Therap* or spiritual or therapeutic touch* or Tovertafel or validation therap* or Video Respite or Virtual Reality or visual feedback* or Yoga or  namaste care).ti,ab,kf. |  |
| --- | --- | --- |
| 7 | Qualitative research/ or exp interview/ or ethnology/ or grounded theory/ or phenomenology/ or exp discourse analysis/ or  exp thematic analysis/ or exp content analysis/ or ethnography/ | 546,814 |
| 8 | (audiointerview* or content analys* or discourse analys* or ethnogeograph* or ethnograph* or ethnolog* or Focus Group* or grounded approach* or Grounded Theor* or Hermeneutic* or Interview* or Narrative Analys* or Naturalistic Inquir* or oral history as topic or Patient Reported or personal narrative* or Phenomenolog* or provider reported or Qualitative or Self Report or Structured Categor* or Thematic Analys* or theoretical sampling* or Unstructured Categor* or  Videointerview).ti,ab,kf. | 1,072,058 |
| 9 | 1 or 2 | 165,117 |
| 10 | 3 or 4 | 452,384 |
| 11 | 5 or 6 | 1,025,901 |
| 12 | 7 or 8 | 1,224,693 |
| 13 | 9 and 10 and 11 and 12 | 820 |
| 14 | limit 13 to english language | 809 |

**APA PsycInfo <1806 to February Week 4 2023>**

| **S. N** | **Search terms combined with Boolean logic using truncation** | **Results f rom 14**  **Mar 2023**  **search** |
| --- | --- | --- |
| 1 | Residential Care Institutions/ or Nursing homes/ or Halfway Houses/ or assisted living/ or Sanatoriums/ or Retirement Communities/ or long term care/ or Group Homes/ | 27,567 |
| 2 | (aged care or Assisted living or care facilit* or care home* or continu* care retirement communit* or convalescenc* home* or convalescenc* hospital* or geriatric* home* or group home* or half way house* or Halfway House* or health service* for the aged or home* for the aged or home* for the elder* or Housing for Older Person* or housing for the elder* or life care cent* or long term care or LTCF or nursing care cent* or nursing home* or old age* home* or old people* home* or Residential Care Institution* or residential facilit* or residential home* or residential institution or respite care or retirement cent* or Retirement Communit* or retirement home* or Sanatorium* or senior residence facilit* or Skilled nursing facilit* or Community living cent* or community cent* or adult family home* or memory care setting).ti,ab. | 34,864 |
| 3 | Dementia/ or Alzheimer's Disease/ | 84,364 |
| 4 | (Alzheimer* or amentia* or dement* or pseudodementia).ti,ab. | 115,383 |
| 5 | exp psychotherapy/ or Auditory Stimulation/ or Massage/ or exp alternative medicine/ or exp Feedback/ or exp Behavior modiﬁcation/ or Bibliotherapy/ or Crisis Intervention/ or Horticulture Therapy/ or Milieu Therapy/ or mindfulness/ or Mindfulness-Based Interventions/ or Catharsis/ or Cognitive Behavior Therapy/ or Mind Body Therapy/ or meditation/ or Cognitive Remediation/ or Sensory Deprivation/ or Hearing Aids/ or Occupational therapy/ or Speech Therapy/ | 374,834 |
| 6 | ("Acceptance and Commitment Therap*" or acoustic stimul* or activit* engagement or Acupressure or Acupuncture or anger management or animal assisted therap* or animal facilitated therap* or applied behavio* analys* or aroma therap* or Aromatherap* or art gallery or art treatment* or Art* therap* or auditory stimul* or Autogenic Training* or behavio* therap* or behavio* treatment* or Bibliotherap* or Cogniti* Remediation or cogniti* rehabilitation or Cogniti* Stimulation* or cogniti* therap* or cognitive enhancement* or Cognitive Intervention* or dance movement or Dance Therap* or Distraction or drama therap* or Environmental modiﬁcation* or Environmental Therap* or ergotherap* or Exercise Intervention* or exercise therap* or Exposure Therap* or eye movement desensitization or family intervention* or Family Therap* or garden* therap* or Gardening or group therap* or group treatment* or Guided Imagery or Hearing aid* or hearing rehabilitation* or horticultur* therap* or hypnos* or life story book* or light therap* or magic table* or Massage or Meditation* or Memory training* or Milieu Therap* or mindfulness or Montessori activit* or multicomponent intervention* or multisensory environment* or multisensory therap* or Music or nonpharmacolog* or non-pharmacolog* or occupation* therap* or Outdoor activit* or person cent* or Pet Therap* or physical activit* or Physical exercise* or physiotherapy or Prayer or Psychotherap* or reality therap* or recreation* therap* or relaxation therap* or Reminiscence* or sensory environment* or sensory stimul* or Simulated presence or snoezelen or social interaction* or Spaced retrieval or Speech Therap* or spiritual or therapeutic touch* or Tovertafel or validation therap* or Video Respite or Virtual Reality or visual feedback* or Yoga or namaste care).ti,ab. | 440,839 |
| 7 | exp Qualitative research/ or exp Interviews/ or ethnology/ or exp phenomenology/ or ethnography/ or Hermeneutics/ | 67,028 |
| 8 | (audiointerview* or content analys* or discourse analys* or ethnogeograph* or ethnograph* or ethnolog* or Focus Group* or grounded approach* or Grounded Theor* or Hermeneutic* or Interview* or Narrative Analys* or Naturalistic Inquir* or oral history as topic or Patient Reported or personal narrative* or Phenomenolog* or provider reported or Qualitative or Self Report or Structured Categor* or Thematic Analys* or theoretical sampling* or Unstructured Categor* or Videointerview).ti,ab. | 632,576 |
| 9 | 1 or 2 | 45,807 |
| 10 | 3 or 4 | 117,619 |
| 11 | 5 or 6 | 637,718 |
| 12 | 7 or 8 | 638,929 |
| 13 | 9 and 10 and 11 and 12 | 387 |
| 14 | limit 13 to english language | 352 |

**CINAHL**

Tuesday, March 14, 2023 4:32:07 AM

| **S. N** | **Search terms combined with Boolean logic using truncation** | **Limiters/Expanders** | **Last Run Via** | **Results(14 March** |
| --- | --- | --- | --- | --- |

|  |  |  |  | **2023)** |
| --- | --- | --- | --- | --- |
| S14 | S9 AND S10 AND S11 AND S12 | Expanders - Apply related words; Apply equivalent subjects Narrow by Language: - english  Search modes -  Boolean/Phrase | Interface - EBSCOhost Research Databases Search Screen - Advanced Search Database - CINAHL  Complete | 1,283(1279  exported) |
| S13 | S9 AND S10 AND S11 AND S12 | Expanders - Apply related words; Apply equivalent subjects Search modes - Boolean/Phrase | Interface - EBSCOhost Research Databases Search Screen - Advanced Search Database - CINAHL  Complete | 1,317 |
| S12 | S7 OR S8 | Expanders - Apply related words; Apply equivalent subjects Search modes - Boolean/Phrase | Interface - EBSCOhost Research Databases Search Screen - Advanced Search Database - CINAHL  Complete | 620,865 |
| S11 | S5 OR S6 | Expanders - Apply related words; Apply equivalent subjects Search modes - Boolean/Phrase | Interface - EBSCOhost Research Databases Search Screen - Advanced Search Database - CINAHL  Complete | 649,828 |
| S10 | S3 OR S4 | Expanders - Apply related words; Apply equivalent subjects Search modes - Boolean/Phrase | Interface - EBSCOhost Research Databases Search Screen - Advanced Search Database - CINAHL  Complete | 109,725 |
| S9 | S1 OR S2 | Expanders - Apply related words; Apply equivalent subjects Search modes - Boolean/Phrase | Interface - EBSCOhost Research Databases Search Screen - Advanced Search Database - CINAHL  Complete | 175,189 |
| S8 | TI ( audiointerview* OR content analys* OR discourse analys* OR ethnogeograph* OR ethnograph* OR ethnolog* OR Focus Group* OR grounded approach* OR Grounded Theor* OR Hermeneutic* OR Interview* OR Narrative Analys* OR Naturalistic Inquir* OR oral history as topic OR Patient Reported OR personal narrative* OR Phenomenolog* OR provider reported OR Qualitative OR Self Report OR Structured Categor* OR Thematic Analys* OR theoretical sampling* OR Unstructured Categor* OR Videointerview ) OR AB ( audiointerview* OR content analys* OR discourse analys* OR ethnogeograph* OR ethnograph* OR ethnolog* OR Focus Group* OR grounded approach* OR Grounded Theor* OR Hermeneutic* OR Interview* OR Narrative Analys* OR Naturalistic Inquir* OR oral history as topic OR Patient Reported OR personal narrative* OR Phenomenolog* OR provider reported OR Qualitative OR Self Report OR Structured Categor* OR Thematic  Analys* OR theoretical sampling* OR Unstructured Categor* OR Videointerview ) | Expanders - Apply related words; Apply equivalent subjects Search modes - Boolean/Phrase | Interface - EBSCOhost Research Databases Search Screen - Advanced Search Database - CINAHL  Complete | 501,799 |

| S7 | (MH "Qualitative Studies+") OR (MH "Interviews+") OR (MH "Ethnology")OR (MH "Ethnological Research") OR (MH "Focus Groups") OR (MH "Phenomenology") OR (MH "Phenomenological Research") | Expanders - Apply related words; Apply equivalent subjects Search modes - Boolean/Phrase | Interface - EBSCOhost Research Databases Search Screen - Advanced Search Database - CINAHL  Complete | 343,356 |
| --- | --- | --- | --- | --- |
| S6 | TI ( "Acceptance and Commitment Therap*" OR acoustic stimul* OR activit* engagement OR Acupressure OR Acupuncture OR anger management OR animal assisted therap* OR animal facilitated therap* OR applied behavio* analys* OR aroma therap* OR Aromatherap* OR art gallery OR art treatment* OR Art* therap* OR auditory stimul* OR Autogenic Training* OR behavio* therap* OR behavio* treatment* OR Bibliotherap* OR Cogniti* Remediation OR cogniti* rehabilitation OR Cogniti* Stimulation* OR cogniti* therap* OR cognitive enhancement* OR Cognitive Intervention* OR dance movement OR Dance Therap* OR Distraction OR drama therap* OR Environmental modiﬁcation* OR Environmental Therap* OR ergotherap* OR Exercise Intervention* OR exercise therap* OR Exposure Therap* OR eye movement desensitization OR family intervention* OR Family Therap* OR garden* therap* OR Gardening OR group therap* OR group treatment* OR Guided Imagery OR Hearing aid* OR hearing rehabilitation* OR horticultur* therap* OR hypnos* OR life story book* OR light therap* OR magic table* OR Massage OR Meditation* OR Memory training* OR Milieu Therap* OR mindfulness OR Montessori activit* OR multicomponent intervention* OR multisensory environment* OR multisensory therap* OR Music OR nonpharmacolog* OR non-pharmacolog* OR occupation* therap* OR Outdoor activit* OR person cent* OR Pet Therap* OR physical activit* OR Physical exercise* OR physiotherapy OR Prayer OR Psychotherap* OR reality therap* OR recreation* therap* OR relaxation therap* OR Reminiscence* OR sensory environment* OR sensory stimul* OR Simulated presence OR snoezelen OR social interaction* OR Spaced retrieval OR Speech Therap* OR spiritual OR therapeutic touch* OR Tovertafel OR validation therap* OR Video Respite OR Virtual Reality OR visual feedback* OR Yoga OR namaste care ) OR AB ( "Acceptance and Commitment Therap*" OR acoustic stimul* OR activit* engagement OR Acupressure OR Acupuncture OR anger management OR animal assisted therap* OR animal facilitated therap* OR applied behavio* analys* OR aroma therap* OR Aromatherap* OR art gallery OR art treatment* OR Art* therap* OR auditory stimul* OR Autogenic Training* OR behavio* therap* OR behavio* treatment* OR Bibliotherap* OR Cogniti* Remediation OR cogniti* rehabilitation OR Cogniti* Stimulation* OR cogniti* therap* OR cognitive enhancement* OR Cognitive Intervention* OR dance movement OR Dance Therap* OR Distraction OR drama therap* OR Environmental modiﬁcation* OR Environmental Therap* OR ergotherap* OR Exercise Intervention* OR exercise therap* OR Exposure Therap* OR eye movement desensitization OR family intervention* OR Family Therap* OR garden* therap* OR Gardening OR group therap* OR group treatment* OR Guided Imagery OR Hearing aid* OR hearing rehabilitation* OR horticultur* therap* OR hypnos* OR life story book* OR light therap* OR magic table* OR Massage OR Meditation* OR Memory training* OR Milieu Therap* OR mindfulness OR Montessori activit* OR multicomponent intervention* OR multisensory environment* OR multisensory therap* OR Music OR nonpharmacolog* OR non-pharmacolog* OR occupation* therap* OR Outdoor activit* OR person cent* OR Pet Therap* OR physical activit* OR Physical exercise* OR physiotherapy OR Prayer OR Psychotherap* OR reality therap* OR recreation* therap* OR relaxation therap* OR Reminiscence* OR sensory environment* OR sensory stimul* OR Simulated presence OR snoezelen OR social interaction* OR Spaced retrieval OR Speech Therap* OR spiritual OR therapeutic touch* OR Tovertafel OR validation therap* OR Video Respite OR  Virtual Reality OR visual feedback* OR Yoga OR namaste care ) | Expanders - Apply related words; Apply equivalent subjects Search modes - Boolean/Phrase | Interface - EBSCOhost Research Databases Search Screen - Advanced Search Database - CINAHL  Complete | 445,906 |
| S5 | TI ( (MH "Psychotherapy+") OR (MH "Massage") OR (MH "Sensory Stimulation+") OR (MH "Aromatherapy") OR (MH "Color Therapy") OR (MH "Mind Body Techniques") OR (MH "Sensory Deprivation") OR (MH "Therapeutic Exercise") OR (MH "Occupational Therapy") OR (MH "Recreational Therapy") OR (MH "Speech Therapy") ) OR AB ( (MH "Psychotherapy+") OR (MH "Massage") OR (MH "Sensory Stimulation+") OR (MH "Aromatherapy") OR (MH "Color Therapy") OR (MH "Mind Body Techniques") OR (MH "Sensory Deprivation") OR (MH "Therapeutic Exercise") OR (MH "Occupational Therapy") OR (MH "Recreational Therapy") OR (MH "Speech Therapy") ) | Expanders - Apply related words; Apply equivalent subjects Search modes - Boolean/Phrase | Interface - EBSCOhost Research Databases Search Screen - Advanced Search Database - CINAHL  Complete | 308,897 |
| S4 | TI ( Alzheimer* OR amentia* OR dement* OR Pseudodement* ) OR AB ( Alzheimer* OR amentia* OR dement* OR Pseudodement* ) | Expanders - Apply related words; Apply equivalent subjects Search modes - Boolean/Phrase | Interface - EBSCOhost Research Databases Search Screen - Advanced Search Database - CINAHL  Complete | 93,657 |

| S3 | (MH "Dementia") OR (MH "Alzheimer's Disease") | Expanders - Apply related words; Apply equivalent subjects Search modes - Boolean/Phrase | Interface - EBSCOhost Research Databases Search Screen - Advanced Search Database - CINAHL  Complete | 78,809 |
| --- | --- | --- | --- | --- |
| S2 | TI ( aged care OR Assisted living OR care facilit* OR care home* OR continu* care retirement communit* OR convalescenc* home* OR convalescenc* hospital* OR geriatric* home* OR group home* OR half way house* OR Halfway House* OR health service* for the aged OR home* for the aged OR home* for the elder* OR Housing for Older Person* OR housing for the elder* OR life care cent* OR long term care OR LTCF OR nursing care cent* OR nursing home* OR old age* home* OR old people* home* OR Residential Care Institution* OR residential facilit* OR residential home* OR residential institution OR respite care OR retirement cent* OR Retirement Communit* OR retirement home* OR Sanatorium* OR senior residence facilit* OR Skilled nursing facilit* OR Community living cent* OR community cent* OR adult family home* OR memory care setting ) OR AB ( aged care OR Assisted living OR care facilit* OR care home* OR continu* care retirement communit* OR convalescenc* home* OR convalescenc* hospital* OR geriatric* home* OR group home* OR half way house* OR Halfway House* OR health service* for the aged OR home* for the aged OR home* for the elder* OR Housing for Older Person* OR housing for the elder* OR life care cent* OR long term care OR LTCF OR nursing care cent* OR nursing home* OR old age* home* OR old people* home* OR Residential Care Institution* OR residential facilit* OR residential home* OR residential institution OR respite care OR retirement cent* OR Retirement Communit* OR retirement home* OR Sanatorium* OR senior residence facilit* OR Skilled nursing facilit* OR Community living cent* OR community cent* OR adult  family home* OR memory care setting ) | Expanders - Apply related words; Apply equivalent subjects Search modes - Boolean/Phrase | Interface - EBSCOhost Research Databases Search Screen - Advanced Search Database - CINAHL  Complete | 148,831 |
| S1 | (MH "Residential Facilities+") OR (MH "Housing for Older Persons") OR (MH "Assisted Living") OR (MH "Long Term Care") | Expanders - Apply related words; Apply equivalent subjects Search modes - Boolean/Phrase | Interface - EBSCOhost Research Databases Search Screen - Advanced Search Database - CINAHL  Complete | 61,417 |

## Appendix 2: JBI data extraction instrument

[https://jbi-global- wiki.reﬁned.site/space/MANUAL/4687826/Appendix+2.3%3A+JBI+Qualitative+data+extraction+tool](https://jbi-global-wiki.refined.site/space/MANUAL/4687826/Appendix%2B2.3%3A%2BJBI%2BQualitative%2Bdata%2Bextraction%2Btool)

## Appendix 3: Template for ConQual summary of findings table

| Appendix 3: ConQual Summary of ﬁndings table | | | | | |
| --- | --- | --- | --- | --- | --- |
| Systematic review title: Factors inﬂuencing the implementation of non-pharmacological interventions for behaviours and psychological symptoms of dementia in residential aged care homes: a systematic review and qualitative evidence synthesis  Population: People living with dementia  Phenomena of interest: non-pharmacological interventions  Context: Residential aged care homes/nursing homes/ long term facilities | | | | | |
| Synthesised Finding | Type of research | Dependability | Credibility | ConQual Score | Comments |
| Insert each synthesised ﬁnding, and complete the columns per synthesised ﬁnding, keeping the rows aligned |  |  |  |  |  |
